# Nanoscale Origins of the Size Effect in the Compression Response of Single Crystal Ni-Base Superalloy Micro-Pillars

**DOI:** 10.3390/ma11040561

**Published:** 2018-04-05

**Authors:** Siqi Ying, Lifeng Ma, Tan Sui, Chrysanthi Papadaki, Enrico Salvati, Leon Romano Brandt, Hongjia Zhang, Alexander M. Korsunsky

**Affiliations:** 1 Department of Engineering Science, University of Oxford, Parks Road, Oxford OX1 3PJ, UK; siqi.ying@eng.ox.ac.uk (S.Y.); t.sui@surrey.ac.uk (T.S.); chrysanthi.papadaki@eng.ox.ac.uk (C.P.); enrico.salvati@eng.ox.ac.uk (E.S.); leon.romanobrandt@eng.ox.ac.uk (L.R.B.); hongjia.zhang@eng.ox.ac.uk (H.Z.); 2 Department of Engineering Mechanics, Xi’an Jiaotong University, Xi’an 710049, China; malf@mail.xjtu.edu.cn; 3 Department of Mechanical Engineering Sciences, University of Surrey, Guildford GU2 7XH, UK

**Keywords:** micropillar, nanoindentation, dislocation, superalloys

## Abstract

Nickel superalloys play a pivotal role in enabling power-generation devices on land, sea, and in the air. They derive their strength from coherent cuboidal precipitates of the ordered γ’ phase that is different from the γ matrix in composition, structure and properties. In order to reveal the correlation between elemental distribution, dislocation glide and the plastic deformation of micro- and nano-sized volumes of a nickel superalloy, a combined in situ nanoindentation compression study was carried out with a scanning electron microscope (SEM) on micro- and nano-pillars fabricated by focused ion beam (FIB) milling of Ni-base superalloy CMSX4. The observed mechanical response (hardening followed by softening) was correlated with the progression of crystal slip that was revealed using FIB nano-tomography and energy-dispersive spectroscopy (EDS) elemental mapping. A hypothesis was put forward that the dependence of material strength on the size of the sample (micropillar diameter) is correlated with the characteristic dimension of the structural units (γ’ precipitates). By proposing two new dislocation-based models, the results were found to be described well by a new parameter-free Hall–Petch equation.

## 1. Introduction

The dependence of the mechanical properties of small material volumes on their size is an important theme in current research, both because it is required to make further progress in design and use of components and structures with ultra-small dimensions (nano-particles, nano-rods, nano-layers) for use in electronics, sensors, catalysis and biomedicine; and because of the need to understand the macroscopic mechanical behaviour of nano-structured materials and composites incorporating these elements. By size, here one can understand either sample dimension (diameter, length, and aspect ratio), or the size of key structural features, such as the grain size, size and spacing of precipitates, etc. One reason for the distinct nature of a deformation response observed in nano-scale objects is due to the proximity of the free surface that can affect both elastic and plastic properties, whilst the other is the fact that the details of interaction between the externally applied load and the internal structure (defects, inhomogeneities, second-phase inclusion etc.) tend to become more prominent under those conditions, and manifest themselves clearly in the stress–strain relationship. 

Size-dependent mechanical behaviour and properties of materials have been studied by various techniques, including micro-cantilever bending, wire torsion, micro-pillar compression [1,2,3,4,5], etc. It has often been observed that apparent material strength changes with the sample size, becoming either greater or smaller, thus manifesting the so-called size effect. The interpretation of such observations requires support from some sort of numerical or conceptual modelling in order to ensure that experimental artefacts are avoided, and the conclusions drawn from the results are soundly justified by the physical mechanisms at work. One common theme in the various analytical approaches concerns the emergence of a characteristic length parameter that can be attributed to structural dimensions (grain size, inclusion size, characteristic length for plasticity, dislocation spacing, etc.). Characteristic lengths can be classified as intrinsic or extrinsic, depending on whether they are inherently present in the material structure, or emerge as a consequence of deformation, as e.g., in the case of strain-gradient plasticity [1,6,7,8].

In the present study, attention was placed on compression testing of micro- or nano-pillars fabricated by focused ion beam (FIB) milling. This method has been applied to a wide variety of materials to reveal a correlation between the observed mechanical strength and the size of the studied material volume [5,9,10,11]. Various explanations have been proposed for the observations, including the widely quoted “dislocation starvation model” that claims that in small-sized objects, higher stress is required to achieve an appreciable density of dislocations that mediate plastic deformation, since they are able to escape through the sample surface. Therefore, mechanical strength is higher in such objects, while mutual interactions are less likely to happen between dislocations, and the secondary strain hardening is absent [1,3,4,12]. 

This approach, however, may not apply for nanostructured materials such as nickel superalloys which contain second-phase inclusions (cuboidal precipitates) even within nano-sized volumes; and in which a high density of dislocation sources is likely to present at the γ/γ’ interfaces, providing ample supply for the onset of slip. On the other hand, evidence has progressively emerged that FIB milling causes material damage to the depths of ~5–30 nm from the milled surface, producing an amorphous layer that may act as an obstacle to dislocation glide, causing additional strengthening [13,14]. In addition, little has been reported on studying the size effect with consideration given to both intrinsic and extrinsic characteristic lengths. These considerations provided the motivation for the present study, together with the obvious practical relevance of nickel supealloys for aerospace engineering, power generation, chemical and oil industries, etc.

In the present study, samples of a single crystal Ni-base superalloy (CMSX-4) were investigated. CMSX-4 is a second-generation, Rhenium-containing nickel-base single crystal superalloy that combines high temperature strength with oxidation resistance, and has been successfully used in numerous aerospace and industrial gas-turbine applications for a quarter of a century, and has been the subject of a wide range of studies [15,16,17,18]. The nominal alloy composition is given in Table 1 [19].

Micro-pillars of various diameters ranging from several microns down to sub-micron dimensions were prepared from single crystals with the <0 0 1> crystallographic direction nominally aligned with the sample surface normal. The alloy possesses a two-phase microstructure that consists of cuboidal γ’ precipitates with the ordered L1_2_ structure embedded in the disordered Face Centered Cubic (FCC) γ phase matrix. The two phases are coherent, in that atomic planes run continuously across the γ and γ’ phases throughout the entire material volume [15]. However, the presence of a small lattice parameter mismatch between γ and γ’ causes straining and distortion of the lattice, resulting in internal stresses that affect the mechanical response of the material, particularly under the conditions of plastic deformation. Dislocations travelling across the crystal meet additional resistance from internal stresses, and encounter obstacles in the form of interphase boundaries that lead to dislocation splitting and increased strength [20]. In addition, the two phases differ in the content of alloying elements (such as Cr) that are preferably segregated to the phase where these atoms can be more readily accommodated, depending on the relative size of the solute atom and the unit cell dimensions [15]. 

**Figure 1:**
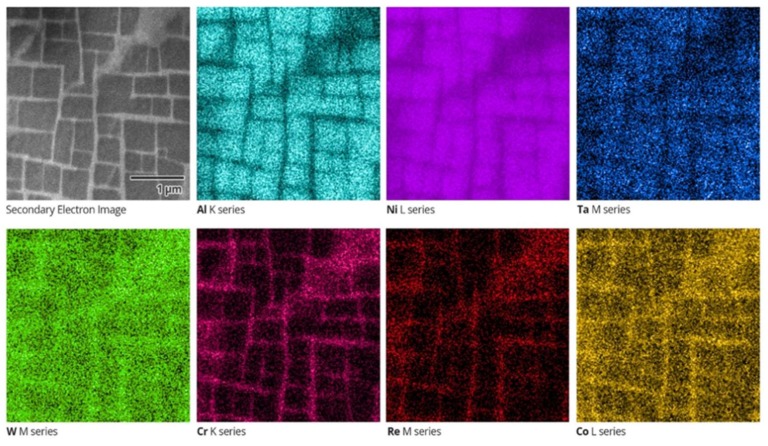
Secondary electron image and energy-dispersive X-ray spectroscopy (EDS) maps of single crystal Ni-base superalloy CMSX-4. The scale of all images is the same as shown in the Secondary Electron Image, and the corresponding elements are indicated.

Figure 1 shows the secondary electron image of a region at the [001]-oriented surface of Ni-base superalloy (note the 1 µm scale bar), along with energy-dispersive spectroscopy (EDS) elemental maps of individual elements over the same area. These were collected using NordlysNano detector (Oxford Instruments, Oxford, UK). Cuboidal precipitates of the reinforcing γ’ phase with the lateral length in the range ~200 nm to ~600 nm appear dark in the secondary electron map, while the matrix γ phase with the typical channel width of 50 nm appears bright. It is apparent from EDS images in Figure 1 that the γ’ phase is rich in Ni, Al and Ta (γ’ phase stabilisers), whilst the γ phase contains increased concentrations of Cr, Co, W and Re (solutes in the γ phase) [15].

The combination of the observed elemental and structural contrast between phases has important implications for the mechanical behaviour of the studied alloy. At room temperature, when dislocation climb is inhibited, the principal mode of deformation is crystal slip involving the continuous travel of dislocations across multiple precipitates. The interphase boundaries, in this case, can be thought of as acting in a way similar to grain boundaries in polycrystals, leading to additional material strengthening. The implications of this hypothesis are tested in the study presented below through the combination of experimental tests and modelling.

## 2. Experimental Method

Micro-pillars for the present study were fabricated using TESCAN LYRA3 FIB-SEM system (TESCAN, Brno, Czech Republic) with a Ga source FIB. A sample of CMSX-4 single crystal with the [001] direction nominally aligned perpendicularly to the surface was ground using a sequence of SiC grinding papers, followed by polishing with 9 μm, 3 μm and 1 μm diamond suspension on polishing cloths. Using FIB ion energy of 30 keV and ion current ~1 nA, annular trenches with the outer diameter ~25 μm were milled around the central micro-pillars with the diameters in the range ~0.5–5 µm. Microscope pre-sets with lower ion current (200 pA and 50 pA) were then applied for slow finishing and polishing of the outside of the pillars. The aim of this operation was to control and reduce the degree of pillar tapering and barrelling, and to reduce the damaged “skin layer” of the micro-pillar samples. This layer arises as a consequence of ion beam damage due to Ga^+^ ions penetrating the material surface, causing a cascade of atomic displacements, creating a population of defects and leading to material amorphisation [21]. This effect has been the subject of a number of recent studies that have primarily focused on silicon, although other materials have also been considered [13,14]. It has been found that alongside the creation of an up to ~30 nm-thick amorphous layer (that presents an obvious obstacle to crystallographic dislocation glide), ion beam damage gives rise to eigenstrain (permanent inelastic strain) and residual stress that may exert additional influence on plastic deformation and strength of these micro-scale test samples. The smallest pillars had sub-micron diameters (down to ~250 nm). These were prepared to obtain sample size that is comparable or smaller than the typical γ’ precipitate dimension (~200–500 nm). At the other end of the scale, the largest pillar diameters extended to ~8 μm to enable reaching the ‘continuum’ limit by studying the response of a material volume containing on average in excess of 250 precipitates. The range of pillar dimensions therefore spanned more than a decade in size.

**Figure 2:**
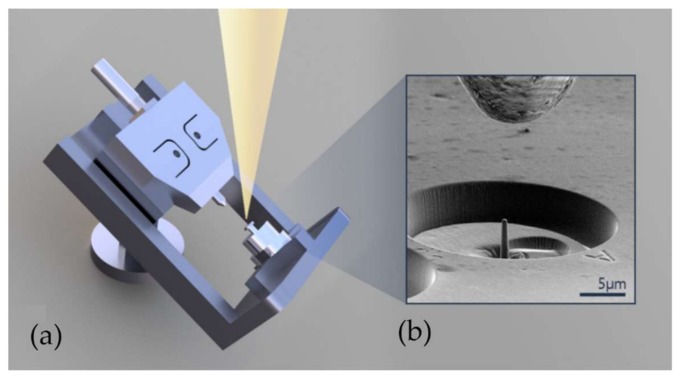
An illustration of the experimental setup: (**a**) pre-tilted frame of the Alemnis in-chamber nanoindenter; (**b**) scanning electron microscope (SEM) image from a 70° tilt angle showing the single crystal diamond flat punch hovering over a focused ion beam (FIB)-fabricated pillar.

Micro-pillar compression experiments were performed using an Alemnis nanoindenter (Alemnis AG, Thun, Switzerland) that was placed inside the FIB–SEM chamber. The overall geometry of the compact mechanical testing platform is illustrated in Figure 2. The indenter consists of a frame on which the motorized sample stage is mounted, together with the indented column that allows nanometer-precision displacement of the diamond tip, whilst the force is recorded at mN resolution. The frame is mounted on the SEM sample stage using a pre-tilted support, allowing SEM image sequence recording during the experiment. The indenter was set to operate in a user-defined displacement-controlled mode that consisted of several experimental stages: alignment, approach, compression, hold, unload and finally the withdrawal of the indenter tip. Alignment and approach were performed either manually, or by choosing the programme tool provided by the manufacturer. A single crystal diamond flat tip punch with the end diameter of ~5 µm was used. The end tip displacement in each experiment was set to achieve the maximum compressive sample strain of ~0.3. Micro-pillar compression stages of the test included a linear displacement ramp to the value corresponding to compressive strain of ~0.3; a holding stage in which the indenter tip remained fixed at the maximum displacement for ~30 seconds; and the retraction stage, for which the same tip displacement ramp rate was used. The indenter tip speed was set to values in the range of 10 nm/s to 50 nm/s depending on the diameter of the pillar. 

Raw load-displacement data were post-processed to identify the point of initial contact with the sample that was assigned zero displacement. Indenter-sample contact was detected by the 20–40% increase of the load with respect to the noise floor that normally lies at the level of ~0.002–0.01 mN. The load drift was removed by a linear fit to the readings prior to sample contact and after unloading. 

An accurate measure of engineering strain $\varepsilon_{eng}$ was obtained by subtracting the elastic response of the indenter and of the bulk material that provides the elastic support below the pillars [4,22]:

(1) $$\varepsilon_{eng}=\left(x_{m}-\frac{1-\upsilon_{i}{}^{2}}{E_{i}}\left(\frac{F_{m}}{D_{t}}\right)-\frac{1-\upsilon_{b}{}^{2}}{E_{b}}\left(\frac{F_{m}}{D_{b}}\right)\right)/H$$

Here $E_{i}$ and$\;\upsilon_{i}$ denote Young’s modulus and Poisson’s ratio of the indenter (1050 GPa and 0.2 for diamond, respectively), and $E_{b}$ and$\;\upsilon_{b}$ denote these parameters for the bulk material (207 GPa and 0.31 for Nickel, respectively). Notation $x_{m}$ and $F_{m}$ is used for the measured displacement and force, respectively, and the top and bottom diameters of the pillar are denoted by $D_{t}$ and $D_{b}$, respectively. H denotes the pillar height. True (logarithmic) strain $\varepsilon_{true}$ and true stress $\sigma_{true}$ were calculated from the engineering parameters $\varepsilon_{eng}$ and $\sigma_{eng}$ using the volume conservation law for the compression mode:

(2) $$\varepsilon_{true}=-\ln\left(1-\varepsilon_{eng}\right)$$

(3) $$\sigma_{true}=\sigma_{eng}\left(1-\varepsilon_{eng}\right)$$

It is clear that these calculations are approximate, as will become apparent from the consideration of the specific geometry of micro-pillar deformation that becomes evident from the image sequences recorded during indentation, as described below. In subsequent analysis, particular focus is placed on the flow stress values that corresponded to particular strain levels read from the stress–strain curves, typically around 0.03 to 0.1 compressive strain. Comparison of the flow stress values between pillars of different diameters was used to obtain insight into the strength size effect in single crystal superalloy micro-pillars.

A sequence of SEM images was recorded in the course of micro-pillar indentation, allowing correlation of distinct deformation phenomena with specific features in the stress–strain plot obtained by the interpretation of the simultaneously recorded load-displacement curve. 

In order to reveal the internal structure of micro-pillars following compression testing, the FIB–SEM dual beam system was used to perform serial sectioning tomography. This was combined with EDS analysis to make use of the additional contrast mode offered by this detection technique. For the purpose of tomographic analysis, the sample was mounted on a SEM stub and the stage was placed at 55° tilt, so that the focused ion beam was incident on the sample perpendicular to the overall sample surface and parallel to the longitudinal axis of the pillar prior to compression. Sample sections were prepared by FIB milling in the polishing regime, and high-resolution SEM images were collected using both secondary electron (SE) and backscattered electron (BSE) detectors. The sample was then moved laterally (translated) by 50 nm perpendicular to the ion beam, and another pair of images recorded. The resulting image stacks recorded in this way allowed volumetric rendering of the internal structure of deformed pillars. 

To achieve a favourable compromise between the efficiency of material removal and the quality of section surfaces, FIB settings used were: accelerating voltage of 30 keV, and ion beam current of 45 pA for section milling, and 1 pA for polishing to achieve the required finish. For the purpose of EDS mapping, the energy dispersive X-ray detector was inserted from the top. The sample stage was tilted further to 70° in order to maximise the EDS signal recorded from the section of the pillar.

## 3. Results

**Figure 3:**
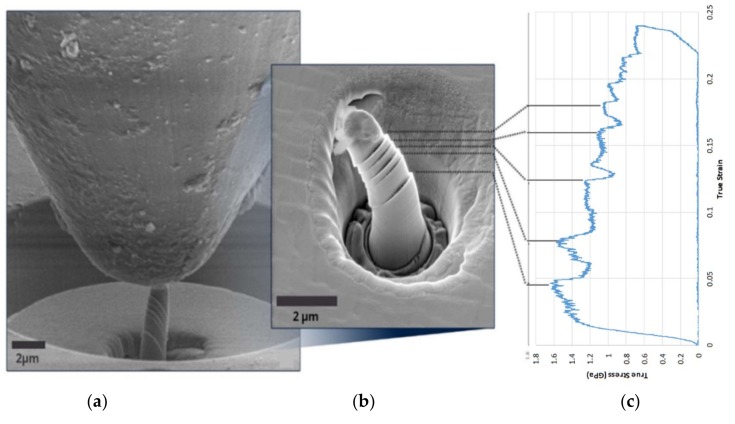
A typical experimental result set: (**a**) a frame taken from a video recorded by SEM at 70° tilt during the compression test of a micro-pillar, showing the large flat-tip indenter at the top, with FIB-machined micropillar within a circular well (bottom middle) undergoing parallel crystal slip; (**b**) zoom of high-resolution SEM image (taken at 30° tilt angle) of a micro-pillar after compression; (**c**) true stress and strain curve of the pillar, with individual stress drops associated with the corresponding distinct slip bands.

A typical set of experimental results is shown in Figure 3. The complete dataset consists of SEM image sequence (video) recording, high-resolution SEM images of the pillar before and after the compression test (see Supplementary Material), and the true stress–strain curve interpreted from the raw load-displacement data captured by the nanoindenter system. The in situ nature of micro-pillar compression and SEM imaging allows direct correlation to be established between deformation phenomena observed in the images, and the features of the load-displacement curve.

**Figure 4:**
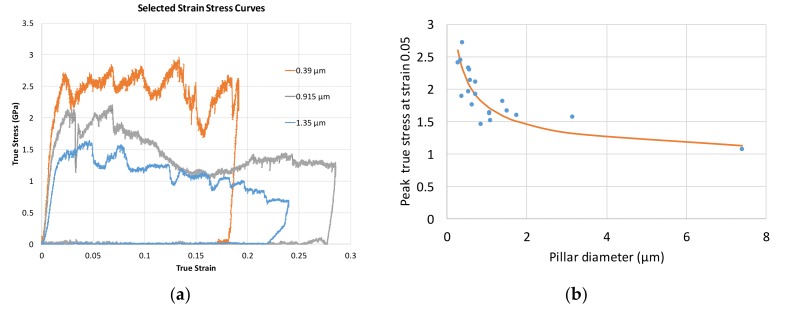
(**a**) Typical stress–strain curves for pillars of different diameters; (**b**) plot of true flow stress at 0.05 true compressive strain for all tested pillars, and fit to the data according to Equation (6).

All recorded stress–strain curves display elastic response at strains below 0.01 (Figure 4a), followed by hardening. At this stage slip traces on the pillar side surface become apparent in the SEM image sequences. There are 12 {111} <$\bar{1}10$> slip systems that can be active in the face-centred cubic crystal structure. The close alignment of the compression axis with the [001] direction means that the normal of the four slip planes of {111} type are inclined at approximately an equal angle of ϕ≅ 54.74°. The same reasoning applies to the slip directions of (011) and (101) types on these slip planes that make the angle λ≅ 35.26° with the loading axis. It is worth noting that slip directions of type (110) are orthogonal to the loading axis, and therefore the critical resolved shear stress on these systems remains close to zero, meaning that no slip can occur. 

The Schmid factor [1,3,4,23] for all eight active slip systems is computed as:

(4) m=cosϕcosλ≅ 0.408

where angle ϕ denotes the angle between the slip plane normal and the loading direction, and λ denotes the angle between the slip direction and the loading direction. 

In real samples used in the experiments, the sample surface normal may not be perfectly aligned with the crystal axis. In accordance with the tolerances of sample preparation and experimental setup the maximum possible misalignment between the loading direction and the [001] orientation of the crystal lattice was estimated not to exceed ~5°.

**Figure 5:**
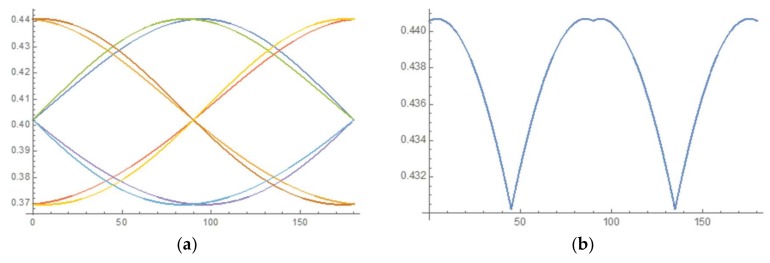
(**a**) Variation of Schmid factor (vertical axis) of the six active slip system as a function of the azimuthal angle of misalignment between the loading direction and the crystal axis [001] in the range 0° to 180° (horizontal axis); (**b**) maximum Schmid factor variation as a function of azimuthal angle in the range 0° to 180°.

Figure 5 presents the plot of Schmid factor variation for the eight active slip systems as a function of the azimuthal angle α of the loading direction with respect to the [001] crystal axis. Since the slip system with the largest Schmid factor value is always activated first, the maximum $\hat{m}$ and minimum $\overset{ˇ}{m}$ values of the maximum Schmid factor are evaluated in this range as:

(5) $$\hat{m}≅0.441\;\overset{ˇ}{m}=0.430$$

In comparison, the Schmid factor for all eight potentially active slip systems in the case of perfect alignment corresponds to the value $\bar{m}≅\;$0.408. In other words, the deviation of the Schmid factor due to 5° misalignment from the value that corresponds to perfect alignment between the loading direction and the [001] crystal axis lies in the range of ~8%. For the misalignment angle of only 1°, this range reduces to less than 2%. Even small misalignment may lead to a significant change in the resolved shear stress on individual slip systems. This leads to one of the slip systems being strongly favoured at the initial stage of loading, which explains that in the experiments the appearance of deformed micro-pillars was dominated by parallel slip. In practical terms this finding means that it is relatively unlikely that cross slip (multiple slip system activation) to occur at the beginning of loading, but it may be caused by subsequent slip system hardening. 

Once plastic deformation begins, the passage of dislocations over parallel slip planes lying close to each other within a slip band leads to self-hardening, i.e., the increase in the shear stress required to cause further slip. Indeed, cross slip is observed in the latter stages of indentation experiments, signifying that the active slip system undergoes hardening of the order of ~10% sufficient to make an alternative slip system more favourable for further slip. 

**Figure 6:**
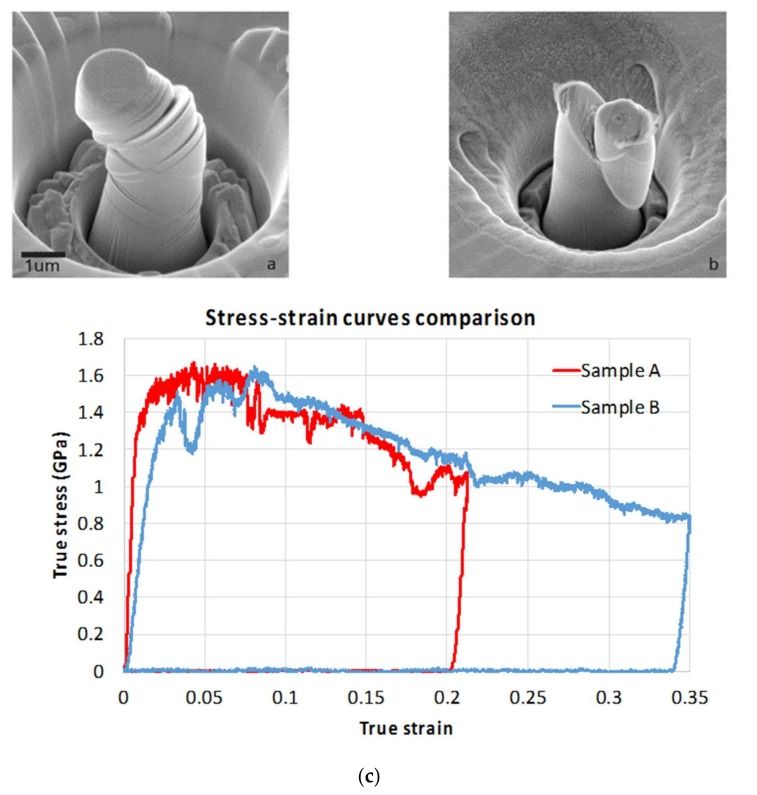
Comparison of two similar sized pillars (**a**) SEM image taken using SE of a 30° tilt 1.5 μm pillar dominated by mutliple cross slip (see dark-light traces visible on the pillar front surface) during compression; (**b**) SEM image of a 30° tilt 1.41 μm pillar dominated by large single slip during compression (same scale bar as Figure a); (**c**) corresponding true stress–true strain traces for the two pillars.

Consideration of the stress–strain plots shown in Figure 4 and Figure 6 reveal that initial Stage I hardening persists up to the strain values of ~0.1. There is an important difference that can be noted between pillars that can be classified as large (~>5 µm) and small (~<2 µm) according to their diameter. For large-diameter pillars the hardening process appears to be continuous and smooth (Figure 4a). This is associated with multiple cross-slip caused by rapid hardening due to the large number of obstacles presented to their glide by interphase boundaries. In contrast, in small pillars the number of interphase boundaries in the dislocation glide path is small, leading to slower increase in the slip resistance within the slip bands. This hardening is insufficient to activate alternative slip systems. Instead, additional slip occurs in parallel slip bands belonging to the same slip system that was activated originally (Figure 3 and Figure 6b).

In the current investigation, in contrast with some of the studies reported in the literature [24,25], displacement control was employed during indentation. In combination with suitably high stiffness and sampling rate of the measurement system, this mode allows efficient detection of load drop whenever the additional slip band is initiated. Figure 6 provides a comparison between the slip geometries observed in two micro-pillars of similar size. The distinction between the two cases demonstrates that the plastic deformation pattern is not size dependent, but is clearly strongly sensitive to small changes in the relative orientation of the crystal with respect to the load axis. What is interesting to note is that at strains in the range 0.03–0.1 the flow stress required for further deformation is consistent between the two cases, suggesting that it may represent a useful parameter for further analysis. It is also apparent that further pillar deformation in Stage II is associated with apparent softening that requires further investigation and explanation. 

**Figure 7:**
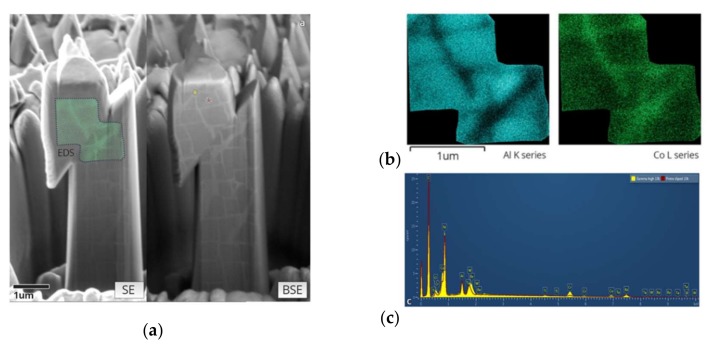
FIB–SEM tomography and EDS results: (**a**) a FIB-milled vertical section of a pillar after compression in both secondary electron (SE) and backscattered electron (BSE) detectors (55° tilt), featured by one slip burst; (**b**) EDS results shows phase contrast by Al and Co mapping, on a selected area indicated on the SE image; (**c**) comparison of EDS spectrum of the typical γ and γ’ phase, indicated on the BSE image.

Figure 7a shows the high-resolution SEM image of a FIB-polished section of a compressed pillar, on both the SE and BSE detectors. The microstructure reveals the arrangement of the γ’ precipitates inside the pillar and how the slip cuts across the material. Large displacement bursts are illustrated by the shearing of the precipitates, and small multiple slips can be seen from the distortion of the precipitate from a rectangular (lower part of the pillar) into parallelogram shape appearing in the upper part of the pillar. The EDS spectrum in Figure 7c is taken to confirm the difference between γ and γ’ phases. It agrees with previous results that the γ phase has a higher concentration of Co, Cr and Re, and the γ’ phase contains more Ni and Al. An EDS elemental mapping of Al and Co over the slip area (indicated in Figure 7a) shows agreement with the same result revealed by the BSE image. 

In the case shown in Figure 7, the consequence of multiple dislocation glide across the pillar is seen in the large steps appearing on the left and right sides of the pillar, and also in the trace of sheared precipitates. This illustrates that dislocations escape before their interaction and multiplication (Stage II hardening) takes place, so that the dislocation density is therefore lower than for bulk samples. According to the popular “dislocation starvation” theory, such relatively dislocation-free status requires a higher shear stress to generate further dislocations, thus leading to material strengthening.

Size effects of the strain hardening of the pillars arises in the present studies as expected. Figure 4a shows representative true stress–strain curves for tested pillars with diameter of 0.39 µm, 0.92 µm, 1.35 μm and 7.88 μm. The smaller pillars (0.39 µm, 0.92 µm, 1.35 μm) clearly show a competition between softening and hardening in the stress–strain curve. After the almost linear hardening in Stage I, the flow stress becomes unstable, decreasing due to large dislocation bursts, and increasing again due to work-hardening processes inside the pillar. Nonetheless, an obvious increase of the strength and work-hardening of [001] pillars with the decreasing diameter is seen. This can be seen from the graphical representation of the results, in that the flow stress at a certain chosen strain value shows an increase with the decreasing pillar diameter. It is shown in Figure 4b that the flow stress of the pillars at 0.05 strain varies from 0.95 GPa (which is somewhat larger, but close to the theoretical strength of the bulk crystal in the [001] orientation of ~0.8 GPa) up to 2.7 GPa, over three times the value for the bulk material.

The top diameter of the pillars is mostly clear-defined and therefore identified as the correlation parameter for consistency. Due to the FIB fabrication process introduced previously, the pillars possess a 2–3° taper prior to compression, which is similar to the value reported in previous publications [1,3,5]. True stress at strain of 0.05 was chosen for the quantification of the size effect across the population of pillars. In this strain range, most pillars show a steady hardening stage towards a peak value before they undergo softening at a later stage of the compression test. 

In order to describe the observed trend quantitatively, a modified Hall–Petch equation which incorporates both extrinsic and intrinsic length scales is introduced, namely:

(6) $$\sigma \left(D\right)=\sigma_{0}\left[1+c{\left(\frac{d}{D}\right)}^{\frac{1}{2}}\right]$$

Here, $\sigma_{0}$ is the flow stress of the bulk material ($\sigma_{0}\;$= 0.78 GPa), *d* is the length of the intrinsic microstructure feature (for example, it is possible to choose *d* = 0.05 μm as the average width of the γ channels), *D* is the external length dimension (pillar diameter), and *c* stands for the fitting parameter that can be derived from the two-phase deformation model presented in Appendix A. According to this model,

(7) $$c=\frac{\left(\tau_{1}-\tau_{2}\right)}{\tau_{1}\cdot f\left(\lambda \right)}$$

where $\tau_{1}$ and $\tau_{2}$ respectively denote the high and low critical resolved shear stress in a two-phase structure, and f(λ) is a geometric function with a value close to unity. 

Figure 4b shows a satisfactory quality fit of Equation (6) to the experimental data. The value of *c* is found to be around 5.5 for the best agreement with the experimental results.

## 4. Discussion

Results of the current study show clear size-dependent behaviour of FIB-fabricated micro-pillars under compression testing. Stress–strain responses collected in the experiment illustrate that all pillars independent of size show a clear elastic stage immediately upon the onset of loading, followed by a hardening stage that persists for the strains between 0.02–0.03 and up to ~0.05, beyond which softening sets in. This softening behaviour is likely to be associated with the combination of the inability of hardening to maintain the required load, and the failure of volume conservation due to large geometric changes. Softening is thought to be observed due to the low speed (10–50 nm/s) displacement control mode employed. In contrast with some studies [4] where load control was used, the loading mode used in this study made it possible to capture the load fluctuation that accompanies the slipping behaviour. 

The results displayed in Figure 4 reveal the size-dependent mechanical response of micro- and nano-pillars under the uniaxial compression test. The observations assisted by FIB cross-sectioning and EDS mapping, have revealed the dislocation gliding behaviour inside the two-phase microstructure of Ni-base superalloys crystals. Ever since it was introduced in the 1950s, the Hall–Petch equation has been applied and modified in numerous ways [26,27,28,29] trying to capture the size-dependent variation of a material’s properties, known as ‘smaller is stronger’. Most recently, studies [1,4,5] tend to seek for an alternative exponent, other than ½, while fitting the size-effect results. The mechanism behind this, however, has not been fully revealed. In the current study, the exponent of ½ is kept in a modified equation based on Hall–Petch and the result manifests great correlation (*R^2^ =* 1 for current study) between the fitting and the experimental data points. It is also realized in some complex models [26,29] where the proportion of dislocation mean free path length to characteristic size (λ) (along with dislocation source density) is considered in the fitting coefficient. However, it (λ) differs from the relation between intrinsic and extrinsic characteristic lengths which is considered in Equation (6). Although a more detailed study remains to be done while possibly carrying out comparison work, the application of Equation (6) sheds light on dual characteristic length consideration in a way that is complementary to the original Hall–Petch equation format.

## 5. Conclusions

In this study, the mechanical response of micro- and nano-sized samples of nickel-base superalloy CMSX-4 was investigated by in-SEM nanoindentation, i.e., in situ compression testing. An in-chamber Alemnis nanoindenter was employed inside a Tescan LYRA FIB-SEM system, while EDS was employed to assist in the observation of the microstructure. The focus was placed on the dependence of flow stress at a certain strain value (0.05), which was compared and discussed as a function of the sample (micropillar) diameter in the range from ~200 nm to several microns. 

The principal outcome of the study lies in the observation of the effect of sample size on the flow stress of the micro- to nano-scale test object. It was found that the dependence on the pillar diameter can be predicted by considering the relative dimension of the sample and that of the precipitates of the reinforcing γ’ phase. Based on careful analysis of dislocation glide within the sample, a modified Hall–Petch equation was proposed that incorporates both extrinsic and intrinsic characteristic lengths. The introduction of this model led to a satisfactory fit with the experimental data.

## Figures and Tables

**Table 1:** CMSX-4 alloy nominal composition.

Element	Wt %	Element	Wt %
Cr	6.5	Al	5.6
Co	9.6	Ti	1.0
W	6.4	Ta	6.5
Re	3	Hf	0.1
Mo	0.6	Ni	Balance

**Figure A1:**
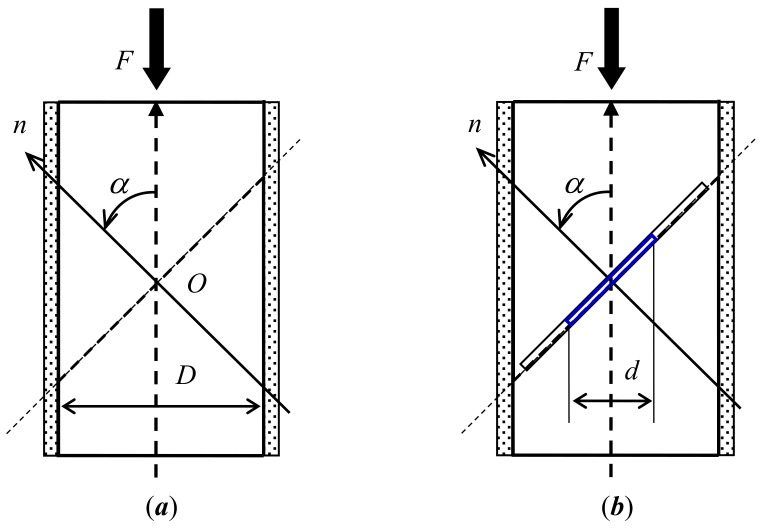
Flow stress of a micro-pillar is under uniaxial compression: boundary and inclusion effect, (**a**) perfect pillar with surface effect; (**b**) equivalent damaged pillar with surface effect, considering the weak phase.

**Figure A2:**
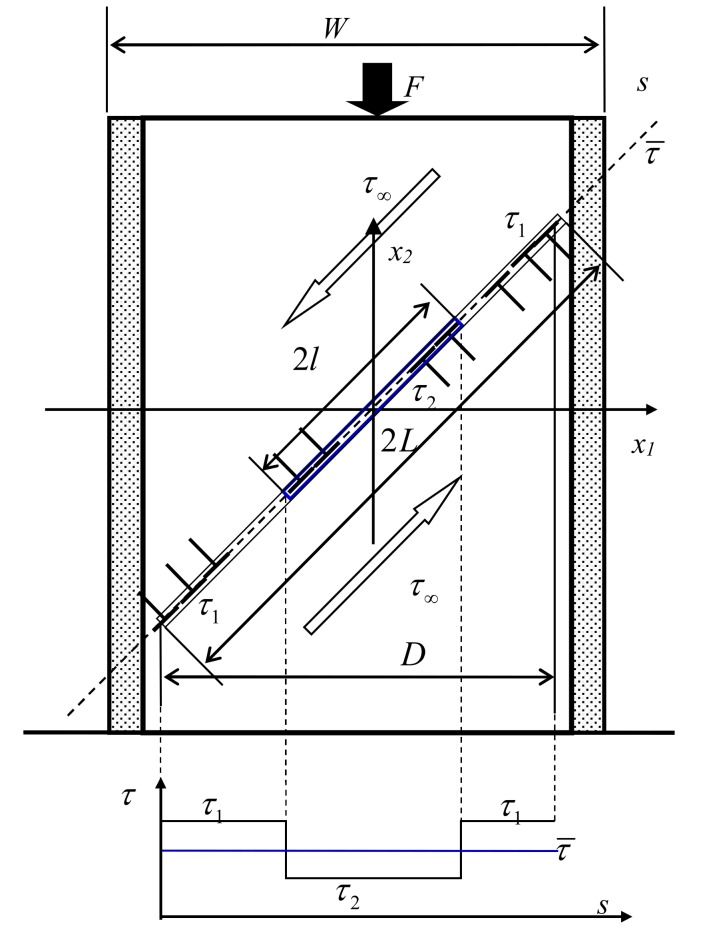
Flow model incorporating the effects of amorphous surface layer and varying internal friction (critical shear stress).

**Figure A3:**
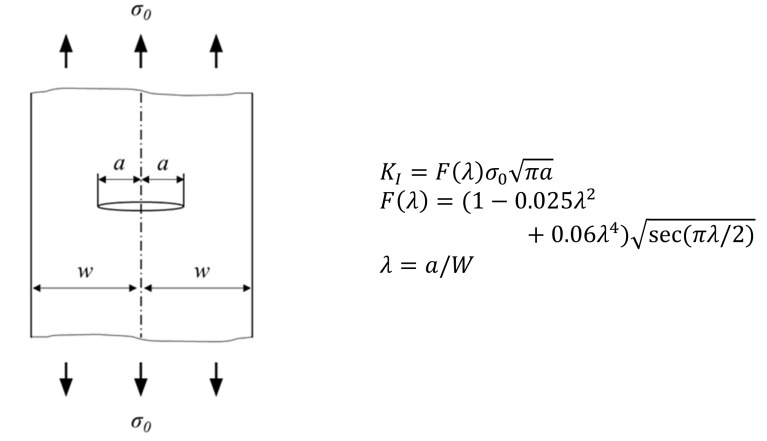
Centre crack in a strip [30].

## Supplementary Materials

Supplementary materials can be found at http://www.mdpi.com/1996-1944/11/4/561/s1.

## Appendix A. Simple Models for Flow Stress of a Two-Phase Single Crystal Pillar under Compression

### Appendix A.1. An Approximate Model Based on the Intrinsic Length Scale d and External Dimension D

Let us assume that on the micro-pillar surface a slip-resistant layer of amorphous material is present, whilst the inner crystalline volume has much lower resistance to slip. The distributed dislocation slip model is illustrated in Figure A1.

This model is shown in more detail in Figure A2.

Under compressive load, a slip band is formed. The internal frictional shear stress for the strong phase is denoted by $\tau_{1}$, whilst the weak phase (matrix) occupies an equivalent slip length 2*l* along the slip band and has a lower friction shear stress denoted by $\tau_{2}$ $\left(\tau_{1}>\tau_{2}\right)$. The overall slip band length is denoted by *2L*, and its average friction shear stress is $\bar{\tau }$. When slip occurs, dislocations of opposite signs travel in opposite directions along the band towards the surface. The overall stress intensity factor due to the dislocation distribution and pile up against the amorphous surface layer is approximately given by:

(A1) $$\begin{array}{l} K_{IIL}=\tau_{macr}\sqrt{\pi L}=\left(\tau_{\infty }-\bar{\tau }\right)\sqrt{\pi L}\\ \bar{\tau }=\frac{2l\tau_{2}+\left(2L-2l\right)\tau_{1}}{2L}=\frac{l\left(\tau_{2}-\tau_{1}\right)}{L}+\tau_{1} \end{array}$$

Now consider the stress intensity factor of the internal slip band that is shown in Figure A2 that arises due to internal dislocation pile up against interphase boundaries, which is given by:

(A2) $$K_{IIl}=\tau_{micr}\sqrt{\pi l}=\left[\tau_{\infty }-\left(\tau_{2}-\bar{\tau }\right)\right]\sqrt{\pi l}$$

In order for the dislocation glide to occur along the entire slip band and to produce an extrusion/intrusion at the pillar surface, the key assumption that this requires equality between slip resistances on the micro- and macro- scale is introduced:

(A3) $$K_{IIL}=K_{IIl}$$

This means that,

(A4) $$\left(\tau_{\infty }-\bar{\tau }\right)\sqrt{\pi L}=\left[\tau_{\infty }-\left(\tau_{2}-\bar{\tau }\right)\right]\sqrt{\pi l}$$

so that,

(A5) $$\begin{array}{l} \left(\tau_{\infty }-\bar{\tau }\right)\sqrt{L}=\left[\tau_{\infty }-\left(\tau_{2}-\bar{\tau }\right)\right]\sqrt{l}=\left[\tau_{\infty }+\left(\bar{\tau }-\tau_{2}\right)\right]\sqrt{l}=\left(\tau_{\infty }+\tau_{l}\right)\sqrt{l}\\ \left(\bar{\tau }-\tau_{2}\right)=\frac{\left(L-l\right)\left(\tau_{1}-\tau_{2}\right)}{L}=\tau_{l}>0 \end{array}$$

From (A5), the following can be found:

(A6) $$\tau_{\infty }=\frac{\sqrt{L}}{\left(\sqrt{L}-\sqrt{l}\right)}\bar{\tau }\left(1+\frac{\tau_{l}}{\bar{\tau }}\frac{\sqrt{l}}{\sqrt{L}}\right)=\frac{\sqrt{L}}{\left(\sqrt{L}-\sqrt{l}\right)}\bar{\tau }\left(1+\frac{\tau_{l}}{\bar{\tau }}\frac{\sqrt{l}}{\sqrt{L}}\right)$$

or

(A7) $$\tau_{\infty }=\frac{\sqrt{L}}{\left(\sqrt{L}-\sqrt{l}\right)}\left(\left(\frac{l\left(\tau_{2}-\tau_{1}\right)}{L}+\tau_{1}\right)+\frac{\left(L-l\right)\left(\tau_{1}-\tau_{2}\right)}{L}\frac{\sqrt{l}}{\sqrt{L}}\right)$$

Furthermore, for the case of moderate *l/L* ratio (e.g., l/L<0.1), Equation (A7) can be simplified as:

(A8) $$\tau_{\infty }=\frac{\sqrt{L}}{\left(\sqrt{L}-\sqrt{l}\right)}\left(\left(\frac{l\left(\tau_{2}-\tau_{1}\right)}{L}+\tau_{1}\right)+\frac{\left(L-l\right)\left(\tau_{1}-\tau_{2}\right)}{L}\frac{\sqrt{l}}{\sqrt{L}}\right)=\tau_{1}\left(1+\frac{\left(\tau_{1}-\tau_{2}\right)}{\tau_{1}}\frac{\sqrt{l}}{\sqrt{L}}\right)$$

Since the values of *l* and *L* are proportional to *d* and *D*, respectively, Equation (A8) can be also expressed in terms of the micro-pillar diameter *D* and intrinsic length scale d as:

(A9) $$\tau_{\infty }=\tau_{1}\left(1+\frac{\left(\tau_{1}-\tau_{2}\right)}{\tau_{1}}\sqrt{\frac{d}{D}}\right)$$

It is noted that all parameters in (A9) are material constants.

### Appendix A.2. A Model Explicitly Incorporating the Finite Size Effect

This is now presented a more detailed model that is built on the previous approach, but incorporates a more complex dependence on the sample size by accounting for the presence of traction-free boundaries.

In Part I, the formula to calculate the stress intensity factor for a slip band (Mode II crack) used the expression for a dislocation distribution in an infinite plane. The finite geometry effect is expressed as follows:

(A10) $$\begin{array}{l} K_{IIL}=\tau_{macr}f\left(\lambda \right)\sqrt{\pi L}=\left(\tau_{\infty }-\bar{\tau }\right)f\left(\lambda \right)\sqrt{\pi L}\\ f\left(\lambda \right)=f\left(\frac{D}{W}\right) \end{array}$$

Here f(λ) is a geometric function that has a form similar to that given by Tada (1971) illustrated in Figure A3.

The stress intensity of the equivalent inner slip band in Figure A2 is still given by:

(A11) $$K_{IIl}=\tau_{micr}\sqrt{\pi l}=\left[\tau_{\infty }-\left(\tau_{2}-\bar{\tau }\right)\right]\sqrt{\pi l}$$

where $\bar{\tau }$ is the average internal frictional stress, and $\tau_{1}$ and $\tau_{2}$ are the internal frictional stresses along the friction line $\left(\tau_{1}>\tau_{2}\right)$. Since,

(A12) $$K_{IIL}=K_{IIl}$$

then,

(A13) $$K_{IIl}=\tau_{micr}\sqrt{\pi l}=\left[\tau_{\infty }-\left(\tau_{2}-\bar{\tau }\right)\right]\sqrt{\pi l}$$

Thus,

(A14) $$\begin{array}{l} \left(\tau_{\infty }-\bar{\tau }\right)f\left(\lambda \right)\sqrt{L}=\left[\tau_{\infty }-\left(\tau_{2}-\bar{\tau }\right)\right]\sqrt{l}=\left[\tau_{\infty }+\left(\bar{\tau }-\tau_{2}\right)\right]\sqrt{l}=\left(\tau_{\infty }+\tau_{l}\right)\sqrt{l}\\ \left(\bar{\tau }-\tau_{2}\right)=\frac{\left(L-l\right)\left(\tau_{1}-\tau_{2}\right)}{L}=\tau_{l}>0 \end{array}$$

From (A14), it follows that,

(A15) $$\tau_{\infty }=\frac{f\left(\lambda \right)\sqrt{L}}{\left(f\left(\lambda \right)\sqrt{L}-\sqrt{l}\right)}\bar{\tau }\left(1+\frac{\tau_{l}}{\bar{\tau }}\frac{\sqrt{l}}{f\left(\lambda \right)\sqrt{L}}\right)$$

or

(A16) $$\begin{array}{ll} \tau_{\infty } & =\frac{f\left(\lambda \right)\sqrt{L}}{\left(f\left(\lambda \right)\sqrt{L}-\sqrt{l}\right)}\left(\bar{\tau }+\tau_{l}\frac{\sqrt{l}}{f\left(\lambda \right)\sqrt{L}}\right)\\ & =\frac{f\left(\lambda \right)\sqrt{L}}{\left(f\left(\lambda \right)\sqrt{L}-\sqrt{l}\right)}\left(\frac{l\left(\tau_{2}-\tau_{1}\right)}{L}+\tau_{1}+\frac{\left(L-l\right)\left(\tau_{1}-\tau_{2}\right)}{L}\frac{\sqrt{l}}{f\left(\lambda \right)\sqrt{L}}\right) \end{array}$$

Furthermore, for small *l*/*L* Equation (A16) is simplified as:

(A17) $$\tau_{\infty }=\tau_{1}\left(1+\frac{\left(\tau_{1}-\tau_{2}\right)}{\tau_{1}}\frac{\sqrt{l}}{f\left(\lambda \right)\sqrt{L}}\right)$$

Equation (A17) can be also expressed in terms of diameters:

(A18) $$\tau_{\infty }=\tau_{1}\left(1+\frac{\left(\tau_{1}-\tau_{2}\right)}{\tau_{1}}\frac{\sqrt{d}}{f\left(\lambda \right)\sqrt{D}}\right)$$

where *d* and *D* can be found in Figure A1. Therefore, Equation (A18) may be written in a generalized form:

(A19) $$\tau_{\infty }=\tau_{1}\left(1+c\frac{\sqrt{d}}{\sqrt{D}}\right)$$

Now *c* is a constant that needs to be determined by the calibration of f(λ), or from fitting the experimental data.
