# Supplementary figures and images for: Nanoscale Origins of the Size Effect in the Compression Response of Single Crystal Ni-Base Superalloy Micro-Pillars
Source: Materials (Basel). 2018 Apr 5;11(4):561. doi: 10.3390/ma11040561 (PMC5951445; doi:10.3390/ma11040561)

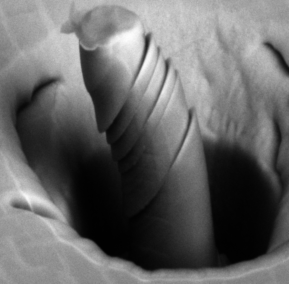

Supplement: Supplementary file 1 [file materials-11-00561-s001.gif]
